# Mechanics of Surface Instabilities in Soft Dielectrics Subject to Electromechanical Loading

**DOI:** 10.3390/polym16243612

**Published:** 2024-12-23

**Authors:** Jiangfei Li, Zehua Wang, Jianyou Zhou

**Affiliations:** School of Science, Harbin Institute of Technology, Shenzhen 518055, China

**Keywords:** soft dielectrics, surface instabilities, wrinkles, creases, electromechanical coupling

## Abstract

As a category of polymeric materials, soft dielectrics, such as most elastomers and rubber-like materials, have shown great potential for extensive applications in various fields. Owing to their intriguing electromechanical coupling behaviors, the morphological instabilities in soft dielectrics have been an active research field in recent years. In this work, the recent progress in experimental and theoretical research on their electromechanical morphological instabilities is reviewed, especially regarding the theoretical aspect. First, we revisit the theoretical framework for the electroelasticity of soft dielectrics. Then, the typical configurations of soft dielectric membranes used to generate two typical types of surface instabilities, namely wrinkles and creases, are introduced. Three commonly used modeling approaches (i.e., the stress balance method, the incremental method, and the energy method) for surface instabilities are reviewed with specific examples. Moreover, discussions on the difference between these methods and the corresponding critical loading conditions are presented. Furthermore, this review also covers the relation and transition between wrinkling and creasing phenomena.

## 1. Introduction

### 1.1. Surface Instabilities

**Figure 1:**
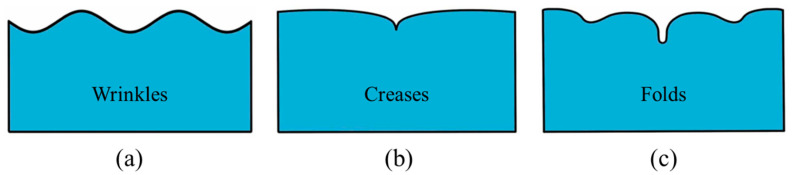
Typical morphologies of surface instabilities: (**a**) wrinkles; (**b**) creases; (**c**) folds.

Soft materials (e.g., biological gels, tissues, and polymeric materials) with low moduli are susceptible to various external stimuli and prone to exhibit surface instabilities, such as wrinkles, creases, and folds (Figure 1). Moreover, compared to the instabilities of hard materials, surface instabilities of soft materials usually result in more complex morphologies and characteristics, which are mainly attributed to their large deformation, material, and geometric nonlinearities [1]. The intriguing surface instabilities of soft materials not only attracted much interest from the research community but also have shown promise in applications in various fields, such as flexible electronics [2,3], substrates for cell growth [4,5], lithography [6,7], microfluidic devices [8,9], and electronic skins [10,11].

**Figure 2:**
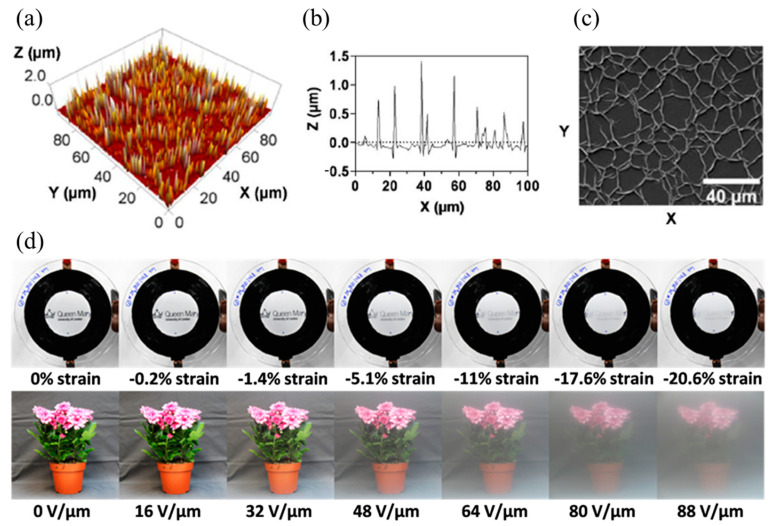
Soft dielectric membrane with tunable surface wrinkles: (**a**,**b**) AFM plot of the surface. (**c**) SEM image of the wrinkles. (**d**) Tuning the transparency of the soft dielectric membrane by changing the applied voltage [14]. Reprinted under the Creative Commons (CC) License (CC BY 4.0).

Furthermore, much effort has been devoted to investigating and exploiting the tunability of such surface instabilities based on soft materials. In the biological field, novel thin films with adjustable surface instabilities can be used not only to create surfaces with super-hydrophilicity, superhydrophobicity, and adjustable wettability [12] but also as dynamic topographical substrates for culturing stem cells [13]. In the architectural field, tunable wrinkles on a soft dielectric membrane can change its transparency and be applied to smart windows in novel buildings (Figure 2) [14]. Soft membranes with tunable wrinkles can also be utilized to fabricate novel optical components, such as tunable optical gratings, lenses, optical diffusers, and more [15,16]. In addition, tunable wrinkles can also be employed to realize special functionalities in soft robots, such as camouflage (stealth) capabilities for reconnaissance and surveillance robots in dynamic environments [17]. Traditional approaches to changing the morphology of wrinkles are mainly based on the idea of altering the strain of the membrane by mechanical stretching or compression. Recently, with the help of actuators based on smart soft materials, such as soft dielectrics, surface instabilities can also be actively tuned by external stimuli (e.g., electric field). Experiments suggest that wrinkles and creases (Figure 1a,b) are the most common surface instabilities observed in soft dielectrics.

### 1.2. Soft Dielectrics

**Figure 3:**
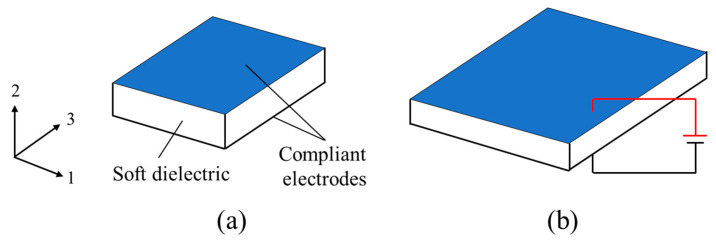
Actuation of a soft dielectric membrane: (**a**) reference state and (**b**) actuated state by a voltage.

With properties of softness (elastic modulus on the order of 10^3^–10^5^ Pa) and lightweight, soft dielectrics can undergo large deformations under the stimulation of an electric field (voltage) [18,19]. Generally, the basic functional unit of a soft dielectric actuator consists of a soft dielectric membrane and compliant electrodes on both sides of the membrane (as shown in Figure 3). When a voltage is applied to the compliant electrodes, positive and negative charges accumulate on the two electrodes, respectively, generating an electrostatic attraction that compresses the soft dielectric membrane in the thickness direction and forces it to expand in the area. The areal strain can be as large as 190% without pre-stretching the material [19]. Due to this electroactive behavior, soft dielectric actuators have extensive potential applications in fields such as soft robotics and biomedical engineering [20,21].

In fact, soft dielectric actuators themselves are susceptible to electromechanical instability (EMI), which is also known as pull-in instability [22]. The EMI can be interpreted as follows. When a soft dielectric membrane is subject to a voltage, the electric field intensifies as the applied voltage rises, causing a rise in the Maxwell pressure that subsequently thins the dielectric membrane. This reduction in the thickness of the membrane, in turn, amplifies the electric field and further elevates the Maxwell pressure, eventually resulting in an excessive thinning of the membrane. EMI can potentially cause premature failure of the dielectric membrane due to electrical breakdown [23]. When the applied electric field surpasses the electrical breakdown strength or dielectric strength of the soft dielectric, electrical breakdown occurs. For the last two decades, various approaches have been proposed to either eliminate the EMI or harness the EMI to produce large deformation [24,25,26].

### 1.3. Methods of Stability Analysis

Different criteria and methods have been established in the literature to determine the stability of a system. The stress balance method is a straightforward method to analyze the stability of a system, which uses stress balance equations to find the maximum or critical point of loading [27,28]. With the stress balance equations, the properties of stability can also be illustrated using the geometric approach [29]. Another commonly used method for stability analysis is the incremental method, which is based on the theory of linearized incremental state variables superimposed on a known state [30,31]. With the balance equations, the incremental version of the boundary-value problem can be obtained, which must result in a zero determinant of the coefficient matrix for the existence of incremental solutions, thus determining the critical state variables.

Furthermore, the energy method is an approach that may simplify the procedure of stability analysis [32,33]. According to the principle of minimum potential energy, the condition for a conservative system to maintain stability is that the total potential energy of the system is at its minimum. In other words, if the total potential energy of the system is the smallest relative to all adjacent states, then the system is stable. Since the total potential energy of a system is a function of the state variables, the determination of stability becomes a mathematical problem of function extremum. In addition, the Hessian approach utilizes the free energy of a system to analyze its stability [34,35]. With the small variations in the state variables, the free energy of the system varies accordingly. Then, the symmetric Hessian matrix is formed by the second-order variations, which must be positive definite if an equilibrium state is stable, thus determining the critical state variables. In fact, other stability criteria are available in the literature, such as the Lyapunov stability criterion [36,37].

## 2. Electroelasticity of Soft Dielectrics

### 2.1. Kinematics

**Figure 4:**
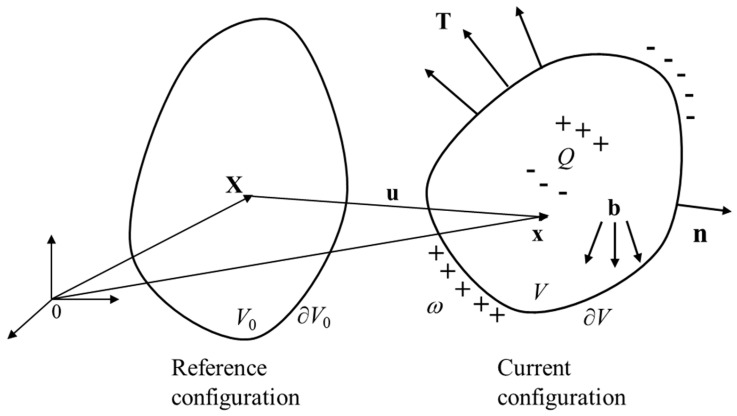
Deformation of a soft dielectric from the reference configuration to the current configuration when subject to electromechanical loading.

Figure 4 illustrates the deformation of a soft dielectric (with mass density *ρ*) from its reference (or original) configuration to its current (or deformed) configuration in response to mechanical forces (surface traction **T** and body forces **b**) and electrical stimuli (free charge per unit volume *Q* and free charge per unit area *ω*). *V*, ∂V, $V_{0}$, and $\partial V_{0}$ denote the volume and the boundary of the current and reference configuration, respectively. In addition, **X** denotes the position of a specific material particle in the reference configuration (the material point), whereas **x** represents the position of the same material particle in the current configuration (the spatial point). The deformation can be described by a smooth function χ as:

(1) $$x=\chi \left(X\right)=u\left(X\right)+X$$

which is called the Lagrangian formulation.

Then, the deformation gradient is defined as:

(2) $$F=\frac{\partial x}{\partial X}=\frac{\partial u}{\partial X}+I$$

where **I** is the second-order identity matrix. Hence, the volume change in the dielectric body is given as $J=\det\left(F\right)$ [38]. Accordingly, the transformation of the unit volume and surface between the current and reference configuration is expressed as:

(3a) $$dV=JdV_{0}$$

(3b) $$dAn=JdA_{0}F^{-T}\cdot n_{0}$$

where dV, dA, **n**, $dV_{0}$, $dA_{0}$, and **n**_0_ are the unit volume, unit area, and unit normal in the current and reference configuration, respectively [39]. In addition, the left and right Cauchy Green deformation tensors are defined, respectively, as $B=FF^{T}$ and $C=F^{T}F$.

### 2.2. The Maxwell Equations

According to the Maxwell equations, the electric field must be curl-free, namely,

(4) curl E=0

where E=∂ϕ/∂x is the true electric field and ϕ is the electric potential of the dielectric body. Furthermore, the divergence of the true electric displacement **D** must equal the free charge per unit current volume *Q*, resulting in:

(5) div D=Q

The electric displacement is also related to the electric field and the true polarization field $\bar{P}$ by:

(6) $$D=\varepsilon_{0}E+\bar{P}$$

where $\varepsilon_{0}$ is the permittivity of the vacuum. For linear soft dielectrics, the relation can be simplified as $D=\varepsilon \varepsilon_{0}E$, where ε is the dielectric permittivity of the dielectric material. Experiments suggest that the dielectric permittivity could be affected by a number of factors, such as deformation, temperature, material viscoelasticity, and the thickness of the membrane. However, *ε* is considered a constant in this work for simplicity. In fact, variable dielectric permittivity can be incorporated into the presented framework if needed. On the surface of the dielectric body,

(7) $$\left(D^{+}-D^{-}\right)\cdot n=\omega$$

where the unit normal is directed from the “−” side towards the “+” of the dielectric body. Furthermore, the nominal electric field $\tilde{E}$ and electric displacement $\tilde{D}$ can be obtained by:

(8a) $$\tilde{E}=F^{T}\cdot E$$

(8b) $$\tilde{D}=JF^{-1}\cdot D$$

where $\mathrm{Curl}\;\tilde{E}=0$, $\mathrm{Div}\;\tilde{D}=Q_{0}$, and *Q*_0_ is the free charge per unit reference volume. Here, Curl and Div are the curl and divergence operators with respect to the reference configuration.

### 2.3. Electromechanical Constitutive Equations

The deformation of the soft dielectric is induced by the mechanical and electrical field. The rate of work $\dot{W}$ performed by the mechanical and electrical field to the soft dielectric is given by:

(9) $$\dot{W}=\int_{\partial V_{0}}v\cdot P\cdot n_{0}dA_{0}+\int_{V_{0}}\rho_{0}b_{0}\cdot vdV_{0}+\int_{V_{0}}\phi_{0}{\dot{Q}}_{0}dV_{0}+\int_{\partial V_{0}}\phi_{0}{\dot{\omega }}_{0}dA_{0}$$

where **v** is the velocity vector, **P** is the first Piola–Kirchhoff (nominal) stress tensor. Here, *ρ*_0_, **b**_0_, $\phi_{0}$, and *ω*_0_ are the mass density, body force, electric potential, and free charge per unit area with respect to the reference configuration, respectively. Conservation of energy yields that:

(10) $$\frac{d}{dt}\int_{V_{0}}\rho_{0}\left(u_{0}+\frac{1}{2}v_{i}^{2}\right)dV_{0}={\dot{W}}_{0}-\int_{\partial V_{0}}q_{0}\cdot n_{0}dA_{0}+\int_{V_{0}}\rho_{0}r_{0}dV_{0}$$

where *u*_0_, **q**_0_, and *ρ*_0_*r*_0_ are the internal energy density, the heat flux per unit area, and the rate of the heat supply per unit volume with respect to the reference configuration, respectively. Combining Equations (9) and (10), and the Maxwell’s equations, we obtain:

(11) $$\frac{d\left(\rho_{0}u_{0}\right)}{dt}=P:\dot{F}+\tilde{E}\cdot \dot{\tilde{D}}-\mathrm{Div}q_{0}+\rho_{0}r_{0}$$

In addition, the Helmholtz free energy density *W*_0_ with respect to the reference configuration is related to the internal energy density by:

(12) $$W_{0}=\rho_{0}u_{0}-Ts_{0}$$

where *s*_0_ is the entropy per unit reference volume, and *T* is the temperature. Moreover, the Clausius–Duhem inequality can be expressed as:

(13) $${\dot{s}}_{0}+\frac{1}{T}\mathrm{Div}q_{0}-\frac{q_{0}}{T^{2}}\cdot \mathrm{Grad}T-\frac{\rho_{0}r_{0}}{T}\geq 0$$

Combining Equations (11)–(13), and $\dot{T}=0$, we obtain:

(14) $$\left[-\frac{\partial W_{0}}{\partial F}+P\right]:\dot{F}+\left(-\frac{\partial W_{0}}{\partial \tilde{D}}+\tilde{E}\right)\cdot \dot{\tilde{D}}-\frac{q_{0}}{T}\cdot \mathrm{Grad}T\geq 0$$

According to the Coleman–Noll procedure [40],

(15) $$P=\frac{\partial W_{0}}{\partial F}$$

(16) $$\tilde{E}=\frac{\partial W_{0}}{\partial \tilde{D}}$$

When incompressibility of the material is assumed, $\det\left(F\right)=1$. Essentially, the incompressibility is a constraint of the deformation. In such a case, the Helmholtz free energy density can be expressed as:

(17) $$W_{0}={\hat{W}}_{0}\left(F,\tilde{D}\right)-p\left[\det\left(F\right)-1\right]$$

where *p* is a Lagrange multiplier. Then the inequality (14) is rewritten as:

(18) $$\left[-\frac{\partial {\hat{W}}_{0}}{\partial F}+p\det\left(F\right)F^{-1}+P\right]:\dot{F}+\left(-\frac{\partial {\hat{W}}_{0}}{\partial \tilde{D}}+\tilde{E}\right)\cdot \tilde{D}+\left[\det\left(F\right)-1\right]\frac{dp}{dt}-\frac{q_{0}}{T}\cdot \mathrm{Grad}T\geq 0$$

Similarly, we can obtain:

(19) $$P=\frac{\partial {\hat{W}}_{0}}{\partial F}-p\det\left(F\right)F^{-1}$$

For an ideal dielectric elastomer, the material model can be taken as:

(20) $${\hat{W}}_{0}\left(F,\tilde{D}\right)=W_{s}+\frac{1}{2\varepsilon_{0}\varepsilon \det\left(F\right)}{\left(F\cdot \tilde{D}\right)}^{2}$$

where $W_{s}$ is the strain energy density function. Ideal dielectric elastomers are characterized by a very low degree of crosslinking and deformation far from their extension limits. Substitution of Equation (20) into (16) and (19) gives that:

(21) $$P=\frac{\partial W_{s}}{\partial F}+\frac{1}{\varepsilon_{0}\varepsilon \det\left(F\right)}\left(F\cdot \tilde{D}\right)⊗\tilde{D}-\frac{1}{2\varepsilon_{0}\varepsilon \det\left(F\right)}{\left(F\cdot \tilde{D}\right)}^{2}F^{-T}-p\det\left(F\right)F^{-1}$$

(22) $$\tilde{E}=\frac{1}{\varepsilon_{0}\varepsilon }F\cdot \tilde{D}\cdot F$$

The Cauchy stress σ is written as:

(23) $$\sigma =PF^{T}=\frac{\partial W_{s}}{\partial F}F^{T}+\varepsilon_{0}\varepsilon E⊗E-\frac{\varepsilon_{0}\varepsilon }{2}{\left|Ε\right|}^{2}I-pI$$

with $\det\left(F\right)=1$. When the strain energy density function is specified, the constitutive equations for a soft dielectric subject to electromechanical loading are obtained. There are a number of options for the strain energy density function in the literature [41].

### 2.4. Biaxial Loading

Consider an undeformed DE membrane of length *L* and thickness *H* (Figure 3a). The membrane is then subject to in-plane biaxial mechanical forces ${\bar{T}}_{1}$, ${\bar{T}}_{3}$, and voltage Φ between the compliant electrodes. Due to the external loads, the DE membrane deforms to the current state with lengths *l*_1_, *l*_3_, and thickness *h*. The corresponding deformation gradient is given as

(24) $$F=\left[\begin{array}{ccc} \lambda_{1} & 0 & 0\\ 0 & \lambda_{2} & 0\\ 0 & 0 & \lambda_{3} \end{array}\right]$$

where *λ*_1_, *λ*_2_, and *λ*_3_ are the stretch ratios in 1-, 2-, and 3-direction, respectively. Assuming the incompressibility of the material, $\lambda_{1}\lambda_{2}\lambda_{3}=1$. According to Equation (23), we can obtain:

(25) $$\sigma_{1}-\sigma_{2}+\varepsilon_{0}\varepsilon E_{2}^{2}=\lambda_{1}\frac{\partial W_{s}\left(\lambda_{1},\lambda_{3}\right)}{\partial \lambda_{1}}$$

(26) $$\sigma_{3}-\sigma_{2}+\varepsilon_{0}\varepsilon E_{2}^{2}=\lambda_{3}\frac{\partial W_{s}\left(\lambda_{1},\lambda_{3}\right)}{\partial \lambda_{3}}$$

where $E_{2}=\Phi /\left(H\lambda_{2}\right)$ is the electric field in 2-direction. The top and bottom surfaces are traction-free, which results in $\sigma_{2}=0$. When the neo-Hookean hyperelastic model is chosen as the strain energy density function of the material, we have:

(27) $$W_{s}\left(\lambda_{1},\lambda_{3}\right)=\frac{\mu }{2}\left(\lambda_{1}^{2}+\lambda_{3}^{2}+\lambda_{1}^{-2}\lambda_{3}^{-2}-3\right)$$

where *μ* is the shear modulus. Then, the Cauchy stresses in 1- and 3-direction can be expressed as:

(28) $$\sigma_{1}=\mu \left(\lambda_{1}^{2}-2\lambda_{3}^{-2}\lambda_{1}^{-2}\right)-\varepsilon_{0}\varepsilon {\left(\frac{\Phi }{H}\right)}^{2}\lambda_{1}^{2}\lambda_{3}^{2}$$

(29) $$\sigma_{3}=\mu \left(\lambda_{3}^{2}-2\lambda_{1}^{-2}\lambda_{3}^{-2}\right)-\varepsilon_{0}\varepsilon {\left(\frac{\Phi }{H}\right)}^{2}\lambda_{1}^{2}\lambda_{3}^{2}$$

When taking equi-biaxial loading as an example, we have ${\bar{T}}_{1}={\bar{T}}_{2}=\bar{T}$, $\sigma_{1}=\sigma_{3}=\sigma =\bar{T}\lambda /\left(LH\right)$, and $\lambda_{1}=\lambda_{3}=\lambda$, which leads to:

(30) $$\frac{\bar{T}}{\mu LH}=\left(\lambda -2\lambda^{-5}\right)-{\left(\sqrt{\frac{\varepsilon_{0}\varepsilon }{\mu }}\frac{\Phi }{H}\right)}^{2}\lambda^{3}$$

When the dimensionless mechanical load $\bar{T}/\left(\mu LH\right)$ and the dimensionless electrical load $\Phi \sqrt{\varepsilon_{0}\varepsilon /\mu }/H$ are prescribed, equi-biaxial stretch ratio λ can be determined by Equation (30).

## 3. Electrowrinkling of Soft Dielectrics

### 3.1. Typical Configurations

**Figure 5:**
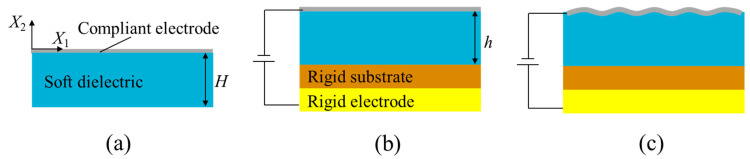
Wrinkles generated by a soft dielectric layer bonded to a rigid substrate: (**a**) Reference configuration of the soft dielectric. (**b**) The soft dielectric is bonded to a rigid substrate. (**c**) Wrinkles are generated by the application of a voltage to the electrodes.

Figure 5 shows the schematic of a widely used configuration with a soft dielectric layer for generating surface wrinkles. The soft dielectric block (with original thickness of *H* and current thickness of *h*) with a compliant electrode on the top surface is bonded to a rigid substrate, and then the substrate is bonded to a rigid electrode. The soft dielectric is sometimes pre-stretched before being fixed on the rigid substrate. The bottom electrode is mostly rigid, while the top electrode is usually compliant. The surface wrinkles are formed when a voltage is applied to the electrodes. For the above configuration, the options for electrode materials are quite extensive, depending on different applications. For instance, van den Ende et al. utilized thin metal films as electrodes to create wrinkles that could reduce light transmittance from 27% to 24% (at a wavelength of 550 nm) [42]. Zang et al. employed graphene as electrodes to successfully generate winkles with superhydrophobicity and high transparency [43]. In addition, in recent years, due to the demand for tunable transmittance films, the research community has begun to explore the use of transparent materials as electrode materials, such as transparent conductive oxides (TCOs) [44,45].

**Figure 6:**
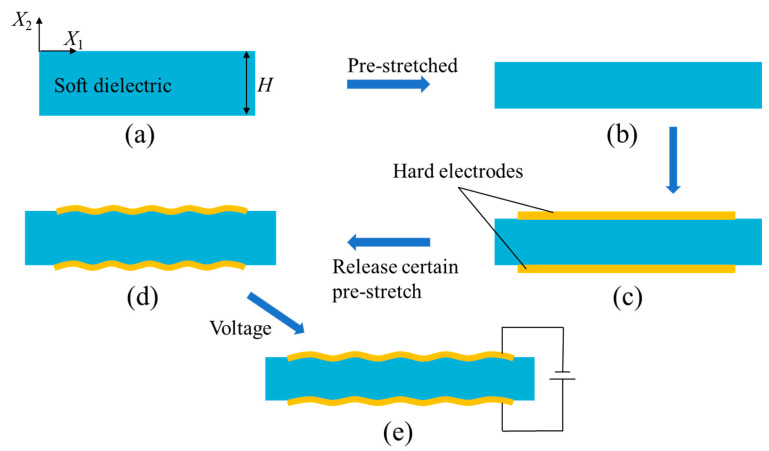
Wrinkles generated by a soft dielectric with two hard electrodes: (**a**) Reference configuration of the soft dielectric membrane. (**b**) Pre-stretched configuration of the soft dielectric membrane. (**c**) Two hard electrodes are bonded to the top and bottom surfaces of the pre-stretched soft dielectric membrane. (**d**) Wrinkles are generated when pre-stretch is released to some extent. (**e**) The wrinkles can be actively tuned when a voltage is applied to the electrodes.

Another commonly used configuration of soft dielectric for generating surface winkles is illustrated in Figure 6. The soft dielectric layer is first pre-stretched (Figure 6b). Then, two hard electrodes are bonded on the top and bottom surfaces (Figure 6c). When the pre-stretch is released to some extent, the hard electrodes wrinkle (Figure 6d). Furthermore, the wrinkles can be flattened as a certain voltage is applied to the electrodes (Figure 6e). With the configuration in Figure 6, Ong et al. utilized transparent indium tin oxide (ITO) films with Young’s modulus of 250 GPa as electrodes, which can withstand up to 25% of biaxial compressive and tensile strain. By tuning the wrinkles of the ITO electrodes with the application of a voltage, the adjustable transmittance range is from approximately 39% to 52% [46]. In addition, highly conductive transparent polymer films have also been developed as electrodes, such as PDMS with carbon black added (Young’s modulus of 910 kPa) [47] and PEDOT:PSS films [48], among others. Lau and co-workers attempted to insert a layer of titanium dioxide film between the PEDOT:PSS film and the dielectric elastomer film, achieving a transmittance range of 1.8% to 81% at a wavelength of 550 nm with minimal compression [49]. However, the lack of accurate electromechanical coupling analysis on the tunable surface wrinkles still hinders their applications.

### 3.2. Electromechanical Wrinkles of Incompressible Soft Dielectric Layers

Considering a 2D formulation, the electromechanical surface instabilities in Figure 5 can be analyzed following the representative works in the literature [50,51,52]. Since the bottom surface is fixed, the plane strain assumption can be taken for simplification. Hence, the deformation gradient is given as:

(31) $$F=\left(\begin{array}{cc} 1+u_{1,1} & u_{1,2}\\ u_{2,1} & 1+u_{2,2} \end{array}\right)$$

Moreover, assuming incompressibility of the material results in det(F)=1, which yields:

(32) $$\frac{\partial u_{1}}{\partial X_{1}}+\frac{\partial u_{2}}{\partial X_{2}}+\frac{\partial u_{1}}{\partial X_{1}}\frac{\partial u_{2}}{\partial X_{2}}-\frac{\partial u_{1}}{\partial X_{2}}\frac{\partial u_{2}}{\partial X_{1}}=0$$

The electric field induced by the applied voltage Φ is expressed as:

(33) $$E={\left(E_{1},E_{2}\right)}^{T}$$

where $E_{1}=0$ and:

(34) $$E_{2}=\frac{\Phi }{h+u_{2}}$$

If pre-stretch is not applied, *h* = *H*.

Adopting the neo-Hookean model [53] as the strain energy density function $W_{s}$, we obtain:

(35) $$\sigma =\mu \left(I+\frac{\partial u_{i}}{\partial X_{j}}+\frac{\partial u_{j}}{\partial X_{i}}+\frac{\partial u_{i}}{\partial X_{j}}\frac{\partial u_{j}}{\partial X_{i}}\right)+\varepsilon_{0}\varepsilon E⊗E-\frac{\varepsilon_{0}\varepsilon }{2}{\left|Ε\right|}^{2}I-pI$$

Without considering the body force and inertia, the equation of motion gives that:

(36) div σ=0

Since the bottom surface is fixed and the top surface is traction-free, the corresponding boundary conditions are:

(37) $$u_{1}\left(X_{1},X_{2}\right)=u_{2}\left(X_{1},X_{2}\right)=0\;\mathrm{at}\;X_{2}=-H,$$

(38) $$P_{12}\left(X_{1},X_{2}\right)=P_{22}\left(X_{1},X_{2}\right)=0\;\mathrm{at}\;X_{2}=0.$$

With Equations from (32) to (38), a boundary-value problem is formed, to which a trivial solution can be obtained as:

(39) $$u=0,\;p=\mu +\frac{\varepsilon_{0}\varepsilon }{2}{\left(\frac{\Phi }{H}\right)}^{2}.$$

When considering small deformation, the boundary-value problem reduces to:

(40) $$\left\{\begin{array}{c} u_{1,1}+u_{2.2}=0,\\ \mathrm{div}\;\sigma =0,\\ \sigma =\mu \left(I+u_{i,j}+u_{j,i}\right)+\varepsilon_{0}\varepsilon E⊗E-\frac{\varepsilon_{0}\varepsilon }{2}{\left|Ε\right|}^{2}I-pI,\\ E_{2}=\frac{\Phi }{h}\left(1-\frac{u_{2}}{h}\right),\\ \begin{array}{l} u_{1}\left(x_{1},x_{2}\right)=u_{2}\left(x_{1},x_{2}\right)=0\;\;\;at\;\;x_{2}=-h\\ \sigma_{12}\left(x_{1},x_{2}\right)=\sigma_{22}\left(x_{1},x_{2}\right)=0\;\;\;at\;\;x_{2}=0. \end{array} \end{array}\right.$$

When the material system is perturbed, the displacement and the Lagrange multiplier can be expressed as:

(41) $$u=u^{0}+u^{*},\;p=p^{0}+p^{*},$$

where “0” and “*” denote the equilibrium value and the small increment of the corresponding quantity. Then, the incremental form of the boundary-value problem (small deformation) becomes:

(42) $$\left\{\begin{array}{c} u_{1,1}^{*}+u_{2,2}^{*}=0,\\ \mathrm{div}\;\sigma^{*}=0,\\ \sigma^{*}=\mu \left(u_{i,j}^{*}+u_{j,i}^{*}\right)+\varepsilon_{0}\varepsilon E^{*}⊗E^{*}-\frac{\varepsilon_{0}\varepsilon }{2}{\left|{Ε}^{*}\right|}^{2}I-p^{*}I,\\ E_{2}^{*}=-E_{2}^{0}\frac{u_{2}^{*}}{h},\\ \begin{array}{l} u_{1}^{*}\left(x_{1},x_{2}\right)=u_{2}^{*}\left(x_{1},x_{2}\right)=0\;\;\;at\;\;x_{2}=-h,\\ \sigma_{12}^{*}\left(x_{1},x_{2}\right)=\sigma_{22}^{*}\left(x_{1},x_{2}\right)=0\;\;\;at\;\;x_{2}=0, \end{array} \end{array}\right.$$

where $E_{2}^{0}=\Phi /h$. Since $u_{1,1}^{*}+u_{2,2}^{*}=0$, $\mathrm{Div}\left(u_{j,i}^{*}\right)=0$. To simplify the calculation, the increment of the electrical stress is omitted here [52,54]. Hence, the equilibrium equation is reduced to:

(43) $$\mathrm{div}\;\left(\mu u_{i,j}^{*}-p^{*}I\right)=\mu u_{i,ji}^{*}-p_{,j}^{*}=0$$

According to the constraint of incompressibility, we can assume that:

(44) $$u_{1}^{*}\left(x_{1},x_{2}\right)=\frac{\partial \psi }{\partial x_{2}},\;u_{2}^{*}\left(x_{1},x_{2}\right)=-\frac{\partial \psi }{\partial x_{1}},$$

where $\psi \left(x_{1},x_{2}\right)$ is a stream function. When a sinusoidal morphology is considered on the top surface of the soft dielectric layer, the following forms of the stream function and Lagrange multiplier can be adopted, i.e.,

(45) $$\psi \left(x_{1},x_{2}\right)=\bar{\psi }\left(x_{2}\right)\cos\left(kx_{1}\right),\;p^{*}\left(x_{1},x_{2}\right)=\bar{p}\left(x_{2}\right)\sin\left(kx_{1}\right).$$

Substitution of Equations (44) and (45) into (42) and (43) yields:

(46) $$\left\{\begin{array}{c} \mu \left(-k^{2}\frac{\partial \bar{\psi }}{\partial x_{2}}+\frac{\partial^{3}\bar{\psi }}{\partial x_{2}^{3}}\right)-k\bar{p}=0,\\ \mu \left(k^{3}\bar{\psi }-k\frac{\partial^{2}\bar{\psi }}{\partial x_{2}^{2}}\right)+\frac{\partial \bar{p}}{\partial x_{2}}=0,\\ \frac{\partial \bar{\psi }}{\partial x_{2}}=\bar{\psi }=0\;\;\;at\;\;x_{2}=-h,\\ \frac{\partial^{2}\bar{\psi }}{\partial x_{2}^{2}}+k^{2}\bar{\psi }=2\mu k\frac{\partial \bar{\psi }}{\partial x_{2}}-\bar{p}-\frac{\varepsilon_{0}\varepsilon {\left(E_{2}^{0}\right)}^{2}}{h}k\bar{\psi }=0\;\;\;at\;\;x_{2}=0. \end{array}\right.$$

Elimination of $\bar{p}$ in Equation (46) results in:

(47) $$\frac{\partial^{4}\bar{\psi }}{\partial x_{2}^{4}}-2k^{2}\frac{\partial^{2}\bar{\psi }}{\partial x_{2}^{2}}+k^{4}\bar{\psi }=0$$

The general solution of Equation (47) is given as:

(48) $$\bar{\psi }=\left(C_{1}+C_{2}x_{2}\right)e^{kx_{2}}+\left(C_{3}+C_{4}x_{2}\right)e^{-kx_{2}}$$

where *C*_1_, *C*_2_, *C*_3_, and *C*_4_ are constant. Substitution of Equation (48) into Equation (46) gives:

(49) $$\bar{p}=2\mu k\left(C_{2}e^{kx_{2}}+C_{4}e^{-kx_{2}}\right)$$

With the boundary conditions in Equation (46), we have:

(50) $$\left(\begin{array}{cccc} e^{-kh} & -he^{-kh} & e^{kh} & -he^{kh}\\ ke^{-kh} & e^{-kh}-khe^{-kh} & -ke^{kh} & e^{kh}+khe^{kh}\\ k & 1 & k & -1\\ \frac{\varepsilon_{0}\varepsilon {\left(E_{2}^{0}\right)}^{2}}{h}-2\mu k & 0 & \frac{\varepsilon_{0}\varepsilon {\left(E_{2}^{0}\right)}^{2}}{h}+2\mu k & 0 \end{array}\right)\left(\begin{array}{c} C_{1}\\ C_{2}\\ C_{3}\\ C_{4} \end{array}\right)=0$$

When the determinant of the coefficient matrix vanishes, the system of linear equations above has a nontrivial solution, which further results in:

(51) $$\frac{\varepsilon_{0}\varepsilon {\left(E_{2}^{0}\right)}^{2}}{\mu }=\frac{2kh\left[e^{4kh}+\left(2+4k^{2}h^{2}\right)e^{2kh}+1\right]}{e^{4kh}-4khe^{2kh}-1}$$

**Figure 7:**
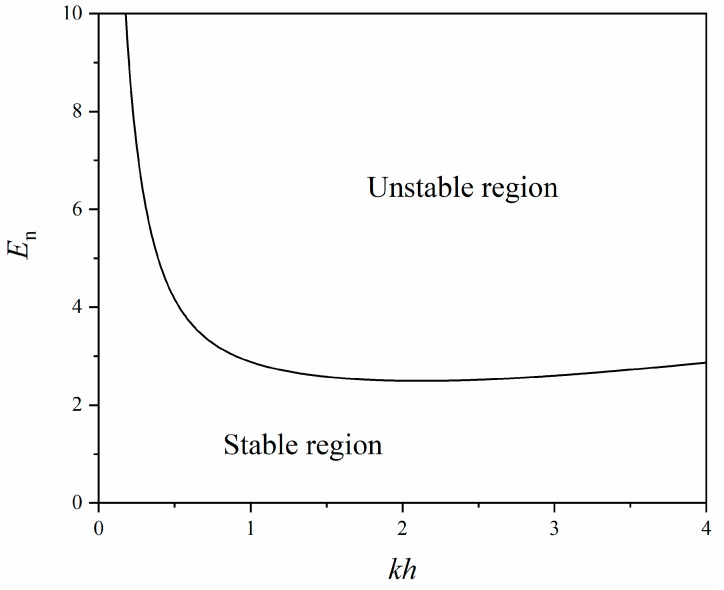
The critical normalized electric field versus *kh*. The region below the *E*_n_ curve is stable, while the region above the curve is unstable.

Figure 7 depicts the normalized critical electric field $E_{n}=E_{2}^{0}\sqrt{\varepsilon_{0}\varepsilon /\mu }$ as a function of ratio *kh*, which has also been explored in previous works in the literature [50,52]. In Figure 7, the region below the obtained curve is the stable region. Moreover, when the normalized electric field is less than the minimum of the curve, surface wrinkling does not occur. In Figure 7, the minimum normalized electric field is about $E_{n}=2.49$ at kh=2.12.

### 3.3. Electromechanical Surface Instabilities of Compressible Soft Dielectric Layers

For compressible soft dielectrics, the Helmholtz free energy density can be expressed as:

(52) $$W_{0}=\frac{\mu }{2}\left(F_{ij}F_{ij}-3-2\ln J\right)+\frac{1}{2}\left(\kappa -\frac{2\mu }{3}\right){\left(J-1\right)}^{2}-\frac{\varepsilon_{0}\varepsilon }{2}JF_{Ki}^{-1}F_{Li}^{-1}E_{K}E_{L}$$

where *κ* is the bulk modulus. Following Landis et al. [55] and considering the perturbation of a homogeneous state (Figure 5b), we have:

(53) $$x_{i}=\delta_{ij}X_{j}+\left(\lambda_{2}-1\right)\delta_{i2}X_{2}+\alpha u_{i}\left(X_{1},X_{2}\right)$$

(54) $$\phi =\frac{\Phi }{H}\left(X_{2}+h\right)+\alpha \varphi \left(X_{1},X_{2}\right)$$

where *λ*_2_ is the stretch ratio in 2-direction, *α* is a dimensionless constant, and $\alpha \varphi \left(X_{1},X_{2}\right)$ is the perturbed electric potential field. At the homogeneous state, the traction-free boundary condition on the top surface leads to:

(55) $$\frac{\varepsilon_{0}\varepsilon }{2}{\left(\frac{\Phi }{H}\right)}^{2}+\left(\kappa -\frac{2}{3}\mu \right)\lambda_{2}^{2}\left(\lambda_{2}-1\right)+\mu \lambda_{2}\left(\lambda_{2}^{2}-1\right)=0$$

Then, the equation of motion (Equation (36)) and the Gauss’ law (Equation (4)) result in:

(56) $$\frac{\Phi }{\lambda_{2}H}u_{2,11}+\frac{\Phi }{\lambda_{2}^{3}H}u_{2,22}-\varphi_{,11}-\frac{1}{\lambda_{2}^{2}}\varphi_{,22}=0$$

(57) $$\left[\left(\kappa -\frac{2}{3}\mu \right)+\mu \left(1+\frac{1}{\lambda_{2}^{2}}\right)\right]u_{2,22}+\mu u_{2,11}+\left[\lambda_{2}\left(\kappa -\frac{2}{3}\mu \right)+\frac{\mu }{\lambda_{2}}\right]u_{1,21}=0$$

(58) $$\left[\lambda_{2}^{2}\left(\kappa -\frac{2}{3}\mu \right)+2\mu \right]u_{1,11}+\mu u_{1,22}+\left[\lambda_{2}\left(\kappa -\frac{2}{3}\mu \right)+\frac{\mu }{\lambda_{2}}\right]u_{2,21}=0$$

The corresponding boundary conditions are:

(59) $$\varphi \left(X_{1},0\right)=0$$

(60) $$\left[\frac{\mu }{\lambda_{2}}-\left(\kappa -\frac{2}{3}\mu \right)\left(\lambda_{2}-1\right)-\frac{\varepsilon_{0}\varepsilon \;\Phi^{2}}{2h\lambda_{2}^{2}}\right]u_{2,1}\left(X_{1},0\right)+\mu u_{1,2}\left(X_{1},0\right)=0$$

(61) $$\begin{array}{l} \frac{\varepsilon_{0}\varepsilon \;\Phi^{2}}{H\lambda_{2}^{2}}\varphi_{,2}\left(X_{1},0\right)+\left[\left(\kappa -\frac{2}{3}\mu \right)+\frac{\mu }{\lambda_{2}}+\frac{\mu {\left(\lambda_{2}-1\right)}^{2}}{\lambda_{2}^{2}}-\frac{\varepsilon_{0}\varepsilon \;\Phi^{2}}{H\lambda_{2}^{3}}\right]u_{2,2}\left(X_{1},0\right)\\ +\left(\kappa -\frac{2}{3}\mu \right)\left(1+\lambda_{2}\right)u_{1,1}\left(X_{1},0\right)=0 \end{array}$$

Assuming a unity wavenumber and small wavelength $\bar{\lambda }$ ($\bar{\lambda }\ll h$), we can obtain the solution of Equations (59)–(61) as:

(62) $$\varphi \left(X_{1},X_{2}\right)=\frac{\Phi }{H\lambda_{2}}\left\{u_{2}\left(X_{1},X_{2}\right)-U_{2}\left(1-A\right)e^{\lambda_{2}X_{2}}\cos\left(X_{1}\right)\right\}$$

(63) $$u_{2}\left(X_{1},X_{2}\right)=U_{2}\left\{e^{X_{2}}-Ae^{BX_{2}}\right\}\cos\left(X_{1}\right)$$

(64) $$u_{1}\left(X_{1},X_{2}\right)=-U_{2}\left\{\frac{1}{\lambda_{2}}e^{X_{2}}-\frac{A\lambda_{2}}{B}e^{BX_{2}}\right\}\sin\left(X_{1}\right)$$

where *U*_2_ is a constant,

(65) $$A=\frac{\varepsilon_{0}\varepsilon {\left(\frac{\Phi }{H}\right)}^{2}+2\lambda_{2}\left[\lambda_{2}\left(\lambda_{2}-1\right)\left(\kappa -\frac{2}{3}\mu \right)-2\mu \right]}{\varepsilon_{0}\varepsilon {\left(\frac{\Phi }{H}\right)}^{2}+2\lambda_{2}\left[\left(\lambda_{2}-1\right)\left(\kappa -\frac{8}{3}\mu \right)+{\left(\lambda_{2}-1\right)}^{2}\left(\kappa -\frac{5}{3}\mu \right)-2\mu \right]}$$

(66) $$B=\lambda_{2}\sqrt{\frac{2\mu +\left(\kappa -\frac{2}{3}\mu \right)\lambda_{2}^{2}}{\mu +\left(\kappa +\frac{1}{3}\mu \right)\lambda_{2}^{2}}}$$

Equations (62)–(64), together with the boundary conditions (59)–(61), form an eigenvalue problem to determine the critical condition for the onset of wrinkling. The critical voltage can be written as:

(67) $$\Phi =H\sqrt{\frac{-b-\sqrt{b^{2}-4ac}}{2a}}$$

where:

(68) $$a=\frac{\varepsilon_{0}^{2}\varepsilon^{2}}{4}\left(1-B\lambda_{2}^{-2}\right)$$

(69) $$b=\varepsilon_{0}\varepsilon \left[\lambda_{2}^{2}\left(\lambda_{2}-1\right)\left(\kappa -\frac{2}{3}\mu \right)-2\mu \lambda_{2}-B\left(\lambda_{2}-1\right)\left(\kappa -\frac{2}{3}\mu \right)+B\left(\lambda_{2}^{2}+\lambda_{2}-1+\lambda_{2}^{-1}\right)\mu \right]$$

(70) $$c=\lambda_{2}^{2}{\left[\lambda_{2}\left(\lambda_{2}-1\right)\left(\kappa -\frac{2}{3}\mu \right)-2\mu \right]}^{2}-B{\left[\lambda_{2}\left(\lambda_{2}-1\right)\left(\kappa -\frac{2}{3}\mu \right)-\left(\lambda_{2}^{2}+1\right)\mu \right]}^{2}$$

**Figure 8:**
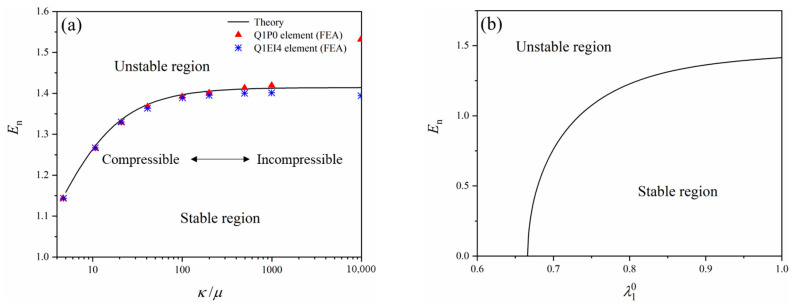
The normalized critical electric field (**a**) as a function of the *κ*/*μ* and (**b**) versus the pre-stretch ratio $\lambda_{1}^{0}$. The FEA data in (**a**) are from the work of Landis et al. [55].

With Equations (55) and (67), the critical voltage can be obtained. The normalized critical field $E_{n}=\Phi \sqrt{\varepsilon_{0}\varepsilon /\mu }/H$ versus κ/μ is shown in Figure 8a. The normalized critical field reaches a plateau of $\sqrt{2}$ as κ/μ increases to a large number (e.g., κ/μ increases to 10,000). In fact, when incompressibility of the material is assumed, κ→∞, B→1, a→0, $b\to 2\varepsilon_{0}\varepsilon \mu$, and $c\to -4\mu^{2}$. Then, the normalized critical electric field in Equation (67) reduces to:

(71) $$E_{n}=\frac{\Phi }{H}\sqrt{\frac{\varepsilon_{0}\varepsilon }{\mu }}=\sqrt{2}$$

From Figure 8a, compressible soft dielectrics are more susceptible to surface instabilities than their incompressible counterparts. Furthermore, the material incompressibility governs the upper bound of the normalized critical field of soft dielectrics. Figure 8a also shows the simulation results from finite element analysis (FEA) with two different types of elements, namely, Q1P0 and Q1EI4 [55].

It should be noted that there is no pre-stretch in the 1-direction ($\lambda_{1}^{0}=1$) before the soft dielectric layer is bonded to the rigid substrate. If pre-stretch or pre-compression $\lambda_{1}^{0}$ is applied, the perturbed solutions (Equation (53)) should be modified as $x_{i}=\lambda_{1}^{0}\delta_{ij}X_{j}+\left(\lambda_{2}-\lambda_{0}\right)\delta_{i2}X_{2}+\alpha u_{i}\left(X_{1},X_{2}\right)$. Then, Equations (53)–(67) should also be altered accordingly. In fact, according to Hutchinson and co-workers [56], considering pre-stretch in 1-direction, the normalized critical electric field at the onset of short wavelength sinusoidal can also be expressed as:

(72) $$E_{n}=\frac{\Phi }{H}\sqrt{\frac{\varepsilon_{0}\varepsilon }{\mu }}=\lambda_{1}^{0}\sqrt{1+{\left(\lambda_{1}^{0}\right)}^{-6}\left[\frac{3-{\left(\lambda_{1}^{0}\right)}^{-3}}{1+{\left(\lambda_{1}^{0}\right)}^{-3}}\right]}$$

Substitution of $\lambda_{1}^{0}=1$ into Equation (72) also results in $\Phi \sqrt{\varepsilon_{0}\varepsilon /\mu }/H=\sqrt{2}$. As shown in Figure 8b, at a pre-compression below $\lambda_{1}^{0}=0.6662$, the mechanical compression can force the soft dielectric layer to wrinkle without the help of the voltage, in which case the Biot’s prediction is reproduced [57]. It has also been found that this critical voltage does not depend on the choice of the free energy function [55].

### 3.4. Electromechanical Creasing Instability

As well established, when the surface of the soft dielectric is compressed, it can bifurcate into different modes of instabilities [58,59]. Other than the commonly seen wrinkles, creases are also typical instabilities [60,61]. Owing to the singular electric field in creases, the linear perturbation analysis is not applicable to creases. Therefore, the energy method is introduced here, as presented in other pioneering works [50]. Since the system tends to maintain minimum potential energy, the critical electrical field for creasing can be determined by comparing the potential energy of the homogeneous state and the creased state. The potential energy $\Pi_{h}$ at the homogeneous state is given by:

(73) $$\Pi_{h}=-\frac{1}{2}\varepsilon_{0}\varepsilon {\left(E_{1}^{0}\right)}^{2}hw+\gamma w$$

where *w* is the width of the elastomer domain and γ is the surface energy. Here, *w* is set to the wavelength of the crease under plane–strain simplification, and γ/μ is defined as the elastocapillary length. It has been found that localized regions on the surface of the soft dielectric layer experience creasing instability when the elastocapillary length is similar to or smaller than the thickness of the layer [50]. Therefore, the transition between wrinkling and creasing can be realized by tuning factors such as surface energy, shear modulus, or the thickness of the soft dielectric layer. Furthermore, the impact of the surface energy highly depends on its sensitivity to defects [62].

For the configuration in Figure 5b, the total potential energy Π of the system is given as:

(74) $$\Pi =U_{E}+U_{M}+U_{S}=-\int_{S}\frac{1}{2}\varepsilon_{0}\varepsilon {\left|E\right|}^{2}dS+\int_{S}W_{s}dS+\int_{l}\gamma dl$$

where *U*_E_, *U*_M_, and *U*_S_ are the strain energy, the electrostatic potential energy, and the surface energy, respectively. Here, *S* is the area of the elastomer domain, and *l* is the contour of the top surface. As shown in Figure 1b, self-contact with a length of $l_{0}$ can be observed when creasing on the surface of the soft dielectric layer happens. From Equation (74), the potential energy of a creased state of the material system can be calculated using the finite element method. According to the works by Chen et al. [62], Wang and Zhao [50], the difference between the homogeneous state and the creased state is given as:

(75) $$\Delta \Pi =\mu l^{2}f\left(E_{2}^{0}\sqrt{\varepsilon_{0}\varepsilon /\mu },l/h\right)+\bar{A}\gamma l$$

where coefficient $\bar{A}=1.81$ is obtained by fitting against results from finite element analysis and $f\left(\cdot \right)$ is a function that governs the potential electromechanical energy difference between the homogenous state and the creased state. The condition for the nucleation of creases is given as ΔΠ=0 and $\partial \Delta \Pi /\partial \left(l/h\right)=0$. Generally, with consideration of the surface energy, the normalized critical electric field for creasing can be approximated as:

(76) $$E_{n}=E_{2}^{0}\sqrt{\frac{\varepsilon_{0}\varepsilon }{\mu }}\approx 1.03+1.88\sqrt{\frac{\gamma }{\mu h}}$$

When the surface energy is neglected, $E_{n}=E_{2}^{0}\sqrt{\varepsilon_{0}\varepsilon /\mu }=1.03$.

### 3.5. Discussions

With specific examples, different methods to determine the critical loading condition of surface instabilities have been reviewed above. The critical electric field calculated from the three examples is also different. In the first example, the critical electric field ($E_{n}=2.49$) is rather high among these cases, which is mainly due to the assumption of small deformation. With consideration of large deformation, the critical electric field is obtained as $E_{n}=\sqrt{2}$ in the second example. Moreover, due to the choice of the strain energy density function (Equation (52)), strain-stiffening is not considered in this case. When creasing is taken into account in the third example, the critical electric field reduces to $E_{n}=1.03$ (surface energy is neglected, and only small deformation is considered). It was found that the critical electric field for creasing was lower than that for wrinkling. However, the calculations above do not include the material viscoelasticity, which leads to time- and rate-dependent behaviors of the soft dielectric. If the material viscoelasticity is considered, the critical electric field could be dynamic.

In fact, in the work of Landis et al. [55], another critical electric field ($E_{n}=1.14$) can be obtained by numerical simulation, which corresponds to the critical electric field of the creased state and is close to the critical value in the second example ($E_{n}=1.03$). When the normalized applied electric field is between 1.14 and 1.41, the soft dielectric layer is stable against small perturbations, while creases may still occur depending on the surface defects or surface energy of the layer. When the normalized applied electric field is beyond 1.41, the soft dielectric layer is unstable against small perturbations, and both creases and wrinkles can nucleate without a barrier. Without considering surface energy, it has been established that creasing, but not wrinkling, is the dominant mode [50,63,64]. In fact, the competition between wrinkling and creasing for compressed elastomeric materials has also been explored in some pioneering works [60,61,65,66]. Nevertheless, due to the indeterminate wavelength and the nonlinear solution path from homogeneous to creased state, numerical analysis of creasing under finite deformation is rather challenging, which could serve as an interesting direction for future study.

Last but not least, although the post-buckling analysis to determine the wavelength and amplitude of wrinkles is not pursued in this work, some general ideas and findings in the literature are given below. In general, equilibrium only predicts the stability condition, while it does not specify the wavelength or amplitude of the wrinkles. Instead, the buckling wavelength (or amplitude) depends on the boundary conditions and can be determined by minimizing the overall free energy of the material system. To find the wavelength and amplitude of the wrinkles, an energetic analysis is commonly adopted [67]. The wavelength is usually a function of the modulus (usually increases with the shear modulus), Poisson’s ratio, and thicknesses of the dielectric layer. Moreover, when the material viscoelasticity of the dielectric layer is considered [68], the wrinkle amplitude grows as time goes on and eventually reaches a steady state.

## 4. Conclusions and Future Study

As soft dielectrics are more frequently used in various fields, this review aims to provide an illustrative glance at the mechanics of their surface instabilities when subject to electromechanical loading. Factors such as the geometric configuration, material parameters, deformations, and electric fields strongly affect the surface instabilities. In this review, the main types of surface instabilities, such as wrinkles and creases that are commonly observed in soft dielectrics, have been revisited. Typical methods for modeling surface instabilities have also been introduced with examples, including the stress balance method, incremental method, and energy method. With the given examples, the effect of the material incompressibility is examined. In addition, the critical loading conditions determined by different methods are compared and discussed. Furthermore, the relation and transition between wrinkling and creasing are also covered in this review.

Despite the notable advancements in recent years in modeling the morphological instability of soft dielectrics, a number of significant and intriguing problems still merit further exploration. Further research on these topics can proceed in different directions. First, polymeric soft dielectrics are more or less viscous [68,69,70], while the viscoelastic effect (originating from the diffusion of the polymer chains) on the instabilities has not attracted much attention. The evolution of the surface morphologies or kinetic surface wrinkling [71,72] under electromechanical loading deserves further experimental and theoretical investigation. Second, the influence of the dielectric properties of soft dielectrics on the surface instabilities is still not well understood. For example, it would be intriguing to explore the current leakage [73,74], deformation and rate-dependent permittivity [75,76], and dielectric strength [77] within this context. Third, in recent years, to improve the actuation performance, a variety of soft dielectric composites have been developed by either adding particles to the dielectric [78,79] or incorporating fibers to reinforce the material [80,81]. The microscale properties of the heterogeneous or anisotropic material could also exert a significant effect on the macroscale surface instabilities. Research on the electromechanical instabilities in heterogeneous or anisotropic soft dielectrics would also be rather helpful in this field. Last but not least, the advances in modeling the surface instabilities in soft dielectrics are significantly affected by the mechanical and mathematical complexities of the practical problems, especially by the interplay among the electromechanical coupling, viscoelasticity, geometric and material nonlinearities, and material properties. Consequently, with consideration of the above-mentioned factors, developing more effective and efficient theoretical frameworks or methods for modeling the surface instabilities of soft dielectrics is of importance in future studies.

## Data Availability

Data are contained within the article.
